# The Cytocidal Spectrum of *Bacillus thuringiensis* Toxins: From Insects to Human Cancer Cells

**DOI:** 10.3390/toxins12050301

**Published:** 2020-05-06

**Authors:** Gretel Mendoza-Almanza, Edgar L. Esparza-Ibarra, Jorge L. Ayala-Luján, Marisa Mercado-Reyes, Susana Godina-González, Marisa Hernández-Barrales, Jorge Olmos-Soto

**Affiliations:** 1 National Council of Science and Technology, Autonomous University of Zacatecas, Zacatecas 98000, Zacatecas, Mexico; grmendoza@conacyt.mx; 2 Academic Unit of Biological Sciences, Autonomous University of Zacatecas, Zacatecas 98068, Zacatecas, Mexico; lesparza@uaz.edu.mx (E.L.E.-I.); marisamercado@uaz.edu.mx (M.M.-R.); 3 Academic Unit of Chemical Sciences, Autonomous University of Zacatecas, Zacatecas 98160, Zacatecas, Mexico; jayala69@uaz.edu.mx (J.L.A.-L.); sgodina@uaz.edu.mx (S.G.-G.); marisahb@uaz.edu.mx (M.H.-B.); 4 Department of Marine Biotechnology, Center for Scientific Research and Higher Education of Ensenada. Ensenada 22860, Baja California, Mexico

**Keywords:** *Bacillus thuringiensis*, Cry, Cyt, parasporins, S-layer proteins, Vip, Sip, membrane receptors, insecticidal activity, anticancer activity

## Abstract

*Bacillus thuringiensis* (Bt) is a ubiquitous bacterium in soils, insect cadavers, phylloplane, water, and stored grain, that produces several proteins, each one toxic to different biological targets such as insects, nematodes, mites, protozoa, and mammalian cells. Most Bt toxins identify their particular target through the recognition of specific cell membrane receptors. Cry proteins are the best-known toxins from Bt and a great amount of research has been published. Cry are cytotoxic to insect larvae that affect important crops recognizing specific cell membrane receptors such as cadherin, aminopeptidase-N, and alkaline phosphatase. Furthermore, some Cry toxins such as Cry4A, Cry4B, and Cry11A act synergistically with Cyt toxins against dipteran larvae vectors of human disease. Research developed with Cry proteins revealed that these toxins also could kill human cancer cells through the interaction with specific receptors. Parasporins are a small group of patented toxins that may or may not have insecticidal activity. These proteins could kill a wide variety of mammalian cancer cells by recognizing specific membrane receptors, just like Cry toxins do. Surface layer proteins (SLP), unlike the other proteins produced by Bt, are also produced by most bacteria and archaebacteria. It was recently demonstrated that SLP produced by Bt could interact with membrane receptors of insect and human cancer cells to kill them. Cyt toxins have a structure that is mostly unrelated to Cry toxins; thereby, other mechanisms of action have been reported to them. These toxins affect mainly mosquitoes that are vectors of human diseases like *Anopheles spp* (malaria), *Aedes spp* (dengue, zika, and chikungunya), and *Culex spp* (Nile fever and Rift Valley fever), respectively. In addition to the Cry, Cyt, and parasporins toxins produced during spore formation as inclusion bodies, Bt strains also produce Vip (Vegetative insecticidal toxins) and Sip (Secreted insecticidal proteins) toxins with insecticidal activity during their vegetative growth phase.

## 1. Introduction

*Bacillus thuringiensis* is a Gram-positive and sporulated bacterium that is widely distributed in soils, plants, and insects around the world [1,2]. Bt is well known because it produces a great variety of useful proteins for pest control in agriculture (Cry, Vip, Sip) [3,4], minimizes diseases transmitted by mosquitoes (Cyt) [5], inhibits pathogens development in animals [6], and because it induces cytotoxicity in human cancer cells (PS, SLP, and Cry) [7,8].

In 1901, Ishiwata found Bt for the first time in *Bombyx mori* and called it *Bacillus sotto*. In 1915, in Thuringia, Berliner isolated this bacterium from the moth *Ephestia kuehniella* and called it *Bacillus thuringiensis* [4].

**Figure 1:**
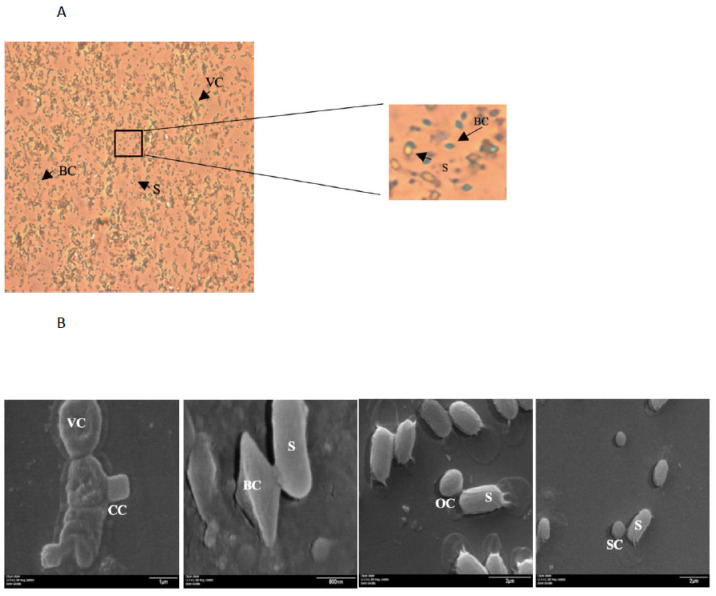
Different morphologies of *Bacillus thuringiensis* crystals; (**A**) image observed at 40× in an optical microscope, the crystal was stained with malachite green. (**B**) Image observed at SEM with different magnifications from the left to the right (1) 20,000×, (2) 15,000×, (3) 15,000×, and (4) 50,000×. VC: vegetative cell; CC: cubic crystal; S: spore; BC: bipyramidal crystal; OC: ovoid crystal; SC: spherical crystal.

During the sporulation process, Bt produces inclusion bodies (IB) in parasporal position with cubic, bipyramidal, spherical, oval, and irregular shapes that can be distinguished by scanning electron microscopy (SEM) (Figure 1) [9,10,11]. The IB are formed by a conglomeration of delta-endotoxin monomers classified according to the sequence similarities between two significant families; Cry and Cyt toxins [10,11,12]. In addition, Bt produces other important toxins such as parasporins, S-layer, Vip, and Sip proteins that will be discussed in this review.

Cry and Cyt proteins received their current nomenclature after creation of the *Bacillus thuringiensis* Toxin Nomenclature Committee [13]. This Committee proposed a classification system of four hierarchical ranks based on the place each toxin occupies in the phylogenetic tree. Cry and Cyt delta-endotoxins with less than 45% sequence identity differ in primary level and are classified as Cyt1, Cry1, Cry2, etc. Cry and Cyt delta-endotoxins with 78% sequence identity differ in secondary rank and a capital letter is added to their name, e.g., Cyt1A, Cry1A, Cry2A. Toxins with 95% identity constitute the border for a tertiary rank and small letters differentiate these proteins from each other, e.g., Cyt1Aa, Cry1Aa, Cry1Ab, Cry1Ac [13,14,15].

Parasporins have less than 25% amino acid sequence homology with Cry toxins [7]. However, their mechanism of action is very similar; both families recognize specific membrane receptors on cancer cells to trigger cell death [6]. Parasporins do not induce hemolytic activity but may or may not have insecticidal activity, nevertheless, they show preferential cytotoxicity against human cancer cells instead of healthy human cells in vitro [16].

Surface layer proteins (SLP) are embedded into cell membranes of many Gram-negative and Gram-positive bacteria; they are commonly associated with polysaccharides and peptidoglycans, respectively [9]. Main functions of SLP proteins are: (1) interaction with extracellular proteins, (2) protection against pathogens, (3) phagocytosis, (4) stabilization of membranes, and (5) adhesion, among others [17]. Unlike *cry* genes, which are expressed during the sporulation process, *s-layer* genes are constitutively expressed throughout the entire cell life cycle. According to previous reports, S-layer proteins from Bt are associated with toxicity against *Epilachna varivestis* [9,18]. Furthermore, there is a report from an S-layer protein with selective cytotoxic activity against MDA-MB-231 breast cancer cells line [8].

Bt synthesizes and secretes to the medium Sip (secreted insecticidal protein) and Vip (vegetative insecticidal protein) proteins during the exponential growth phase. There are reports about the insecticidal activity of these proteins against some coleopterans and lepidopterans [19,20,21].

Bt toxins have been isolated and classified into at least 78 Cry [13], 3 Cyt [13], 6 parasporins [7], 1 SLP [18], 1 Sip [11], and 4 Vip families [11].

## 2. Leading Toxic Proteins of *Bacillus thuringiensis* and their Mechanism of Action

### 2.1. Cry Toxins

Cry proteins are widely known by their toxic activity against insects belonging to orders such as *Hymenoptera*, *Coleoptera*, *Homoptera*, *Orthoptera*, and *Mallophaga*, as well as nematodes, mites, and protozoa [2,11,22,23]. Their toxic activity against insect larvae has allowed these toxins to be used for biological control of pests through spray formulations and transgenic crops that incorporate Cry proteins or some active fragment [22,24,25,26,27,28]. Tobacco was the first Bt crop produced by “Plant Genetic Systems” in Belgium in 1985 [29]. Since then, other crops, such as corn, cotton, potato, rice, brinjal, and soybean, have been genetically modified with Bt toxins to resist insect pests [30,31].

Cry toxins are highly specific to their target organisms. It is unusual to find a Cry toxin that targets more than one insect order, as is the case of Cry1Ba which is toxic to moths, flies, and beetles larvae [32]. *Cry* genes reside on plasmids that are naturally transferred from one Bt strain to another by conjugation or recombination [33,34]. This transfer of information plays an essential role in the biodiversity of Bt strains [34]. The final composition of *cry* genes in a strain determines the specificity and toxicity against biological targets, including human cells [3,15,34]. More than 700 *cry* genes have been classified into groups and subgroups, according to their amino acid sequence similarity [11,13].

X-ray crystallography of Cry proteins has evidenced three structural domains; hence, Cry toxins are also known as 3d-Cry toxins. The N-terminal Domain I is formed by seven α-helices, with a conserved hydrophobic helix α-5 in the center, which is related to oligomerization of the toxin [3,4,35]. Helix α-5 is also responsible for pore-formation in the membrane of susceptible cells, and for toxin insertion into the cell. Given these characteristics, Cry proteins are classified as pore-forming toxins (PFT). In this sense, Domain I is the most conserved among all Cry toxins, sharing some structural similarity with Colicin Ia, another PFT [3,4,36,37].

Domain II is composed of three antiparallel β-sheets that form a hydrophobic core, with highly variable exposed loop regions. This domain is responsible for toxin specificity; therefore, indicates the binding sites into receptors in susceptible larvae [3,38,39,40].

Domain III is composed of antiparallel β sheets that form a β sandwich structure. This domain is also involved in receptor binding specificity; additionally, it is also associated with pore formation in cell membranes [3,38,39,40].

Cry toxins belong to the PFT family due to their mechanism of action, in which Domain I is inserted into the membrane of target cells, creating a trans-membrane ion channel and triggering the host’s death [2,3,41]. The PFT family comprises different types of toxins, including, among others, Colicin family, produced by Escherichia coli [42]. The ClyA family is produced by *Escherichia coli* and *Salmonella* enteric strains [43]. The Actinoporin family is produced by sea anemones [44]. The Haemolysin family, produced by *Staphylococcus aureus* [45]. The Aerolysin family, produced by *Aeromonas hydrophila* [46]. The CDC family, produced by pathogenic Gram-positive bacteria such as *Clostridium perfringens*, *Bacillus anthracis*, and *Streptococcus pneumoniae* [47]. The toxins from the PFT family have several characteristics in common: (1) The way they fold, which suggests all share a similar mechanism of action [48,49,50,51]; (2) All recognize specific receptors on cell membranes [48,49,50,51]; (3) Promote oligomerization at the interaction site after receptor recognition [48,49,50,51].

PFT are classified into two main groups according to their secondary structures, which are responsible to the formation of pores: toxins from α-helical group includes Colicin, Exotoxin A, Diphtheria, and Cry toxins. In this group, the α-helix region is responsible for the trans-membrane ion channel formation [48,49,50]. On the other hand, β-barrel toxins include Aerolysin [50], Hemolysin [45], Perfringolysin O [52], and Cyt toxins [53]. These toxins insert themselves into the cell membrane, forming a barrel composed of β sheets hairpin monomers [48,49,50].

#### Mechanism of Action from Cry Toxins

3d-Cry proteins are produced as large protoxins with a molecular weight around 130 kDa, such as Cry1Aa protein [3,4,11,14], or short protoxins between 65 and 70 kDa, such as Cry11Aa protein [4,14]. The large protoxins are processed by insect midgut proteases at C-terminal and N-terminal ends [3,4,11,14], while short protoxins are processed only at the N-terminal end [4,14]. In both cases, protease-resistant core results in an active Cry toxin of 60 and 70 kDa approximately which retains the 3d structure [1,4,11,14]. The resistant fragment is responsible for cytotoxicity against larvae insects, nematodes, protozoans, and human cancer cells [1,4,11,14]. However, incorrect or deficient protoxin activation, and/or rapid degradation of toxins by other proteases, could reduce the toxicity of Cry proteins against their target [4,14].

##### (a) The Pore-Forming Model

**Figure 2:**
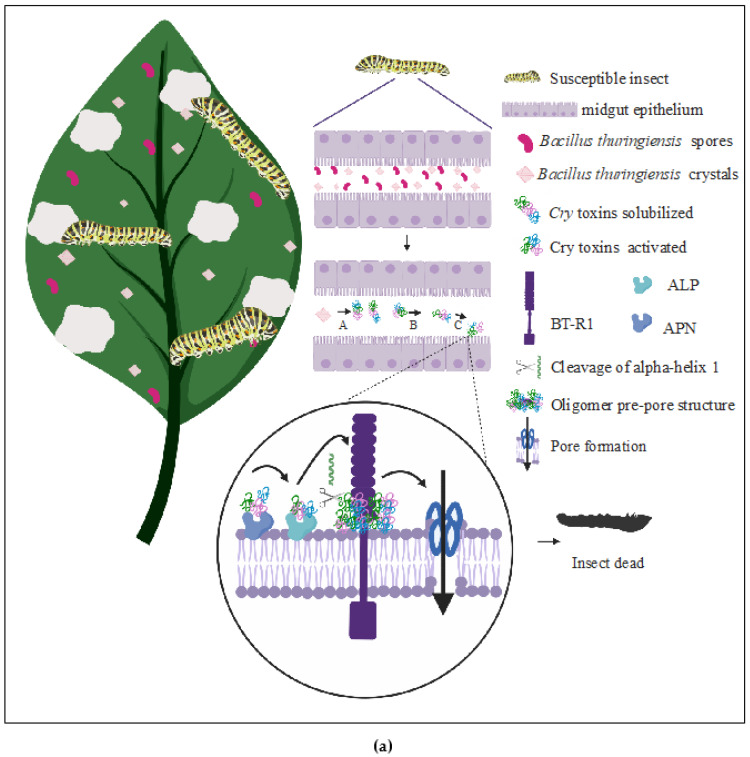

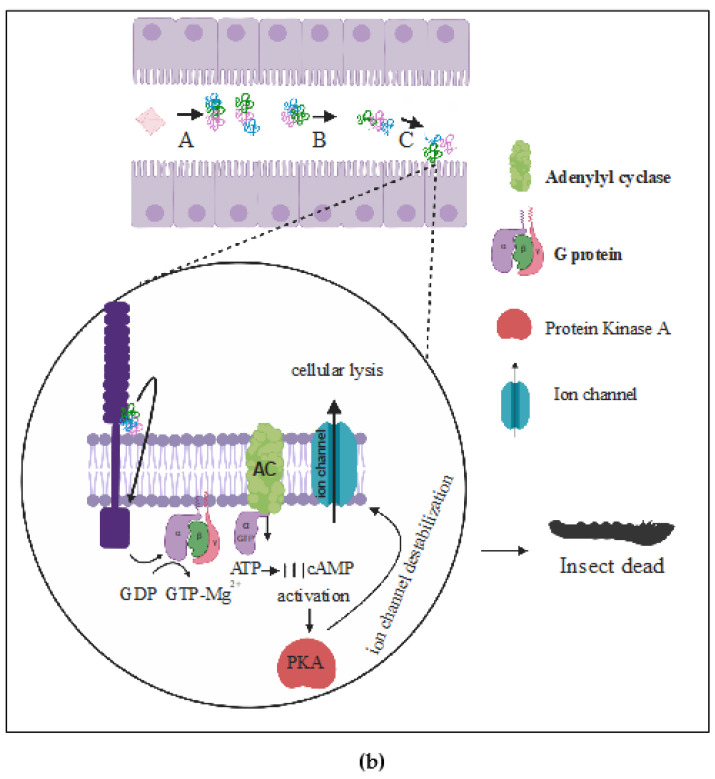
Mechanism of action of Cry proteins. (**a**) Pore-forming model, once larvae ingest crystals, these are solubilized and proteolyzed in larvae midgut. Cry toxins recognize APN, ALP, and EC12/BT-R1 membrane receptors. Cry toxins suffer a proteolytic cleavage on helix α1, resulting in formation of a pre-pore oligomer structure. Posteriorly, oligomer structure is inserted into the cell membrane and creates an ionic pore that leads to osmotic failure, followed by septicemia and insect death. (**b**) Signaling pathway model, once Cry toxins recognize and bind to a cadherin receptor, induces activation of adenylyl cyclase that triggers an increase in cAMP and activates protein kinase A (PKA). This activation will induce a cascade of events that results in an ion channel formation in the membrane, cytoskeleton destabilization, and programmed cell death. A, Solubilization. B, Activation by proteolysis. C, Recognition of membrane receptor. Created with Biorender.com.

The most accepted mechanism of action of Cry toxins against insect larvae is the pore-forming model [33,37,54,55], which is summarized in Figure 2A. Once larvae ingest toxin crystals, these solubilized at extreme pH (acid or alkaline, depending on the Cry toxin) and proteolyzed by proteases under suitable physicochemical conditions on the midgut. The activated toxins can reach the apical brush border membrane (microvilli) of the insect’s midgut by crossing the peritrophic matrix. Cry toxins must recognize receptors in brush-border membranes to form pores; therefore, specificity is crucial for Cry proteins toxicity [3,55,56]. In this sense, Domain I needs to be inserted into the cell membrane through its hydrophobic helical hairpin [55,57]. The amphipathic helices attach to the surface of membranes using hydrophobic helices α-4 and α-5 to enter into the phospholipid bilayer [55,57]. In addition, highly variable and exposed loops from Domain II also participate in binding to receptors, a process that apparently involves two steps. The first step consists of recognition of aminopeptidase N (APN) and alkaline phosphatase (ALP) receptors and the formation of a weak binding with Cry toxins [11,55,56,57,58,59], which produces a reversible reaction [10,55].

The second step consist in the formation of an irreversible binding (Kd 19 nM) through recognition of a 12 amino acid ectodomain region (EC12) from the cadherin receptor (BT-R1) [10,60,61,62]. Conserved sequence motifs near the N and C ends of EC12 have been reported to be crucial for binding of toxins in insect cells [10,61,62]. Bt-R1 is a highly specific and selective binding receptor to Cry1Ab toxin, it was identified for the first time in the midgut of *Manduca sexta* larvae; this receptor is also responsible for Cry1Ab oligomerization [60,61,62].

BT-R1 is a protein of 210 kDa composed of four domains: (1) an ectodomain with twelve modules (EC1-EC12) composed of β-barrel cadherin repeats; (2) a membrane-proximal extracellular domain; (3) a transmembrane receptor; (4) a cytoplasmic domain [10,60,63]. The interaction between Cry1A toxin and BT-R1 facilitate the proteolytic cleavage of helix α1 from Domain I, which is located at the N-terminal end, resulting in the formation of a pre-pore oligomer structure that increases the affinity between oligomer and membrane receptors APN and ALP [35,62,64]. The oligomer inserted into the cell membrane creates an ionic pore that leads to osmotic failure, followed by septicemia and insect death [2,12,23,41]. Other intracellular molecules, such as actin, flotillin, prohibitin, and V-ATPase, have been found to participate in the binding to Cry toxins [11,65].

Domain III is a key structure in toxin stability, it binds to N-acetylgalactosamine (GalNAc) in the APN receptor [55,66,67]. APN has been identified as a binding receptor for Cry1A toxins in *M. sexta*, *H. dispar* [55,66,67], and *B. mori* [55]. ATP-binding cassette transporters (ABC proteins) are also involved in Cry toxicity, especially member 2 of subfamily C (ABCC2). These proteins may help Cry1A toxins carry out their primary task: binding to receptors and inserting oligomers into the cell membrane [68,69].

However, recently published evidence has shown that toxicity of Cry1AbMod and Cry1AcMod (Cry toxins whose structure has been modified by deleting helix α1) against *M. sexta* that does not involve the expression of the cadherin receptor and still can form toxic oligomeric structures [70,71,72]. This finding is of great importance for development of strategies to counteract resistance in transgenic crops, and to increase our knowledge of the mechanism of action of Cry toxins.

##### (b) The Signaling Pathway Model

Cell culture toxicity assays developed to elucidate the action mechanism used by Cry toxins in the signaling pathway model have been carried out in High Five (H5) cell line; which involves expression of a cadherin receptor from *Manduca sexta* [73,74] in a Sf9 cell line from lepidopterans [74,75]. The signaling pathway model postulates that Cry proteins cytotoxicity is mediated by recognizing and binding to a cadherin receptor, which activates an Mg^2+-^ dependent cellular signal cascade pathway that leads to cell death [74]. The binding between Cry toxin and cadherin receptor induces the activation of adenylyl cyclase; which triggers an increase in cAMP and activates protein kinase A (PKA). These events trigger a cascade that results in an ion channel formation on the membrane, cytoskeleton destabilization, and programmed cell death (Figure 2B) [76].

### 2.2. Cyt Family

Cyt toxins size ranges from 25 to 28 kDa; their three-dimensional structure shows Cyt proteins have a single α-β domain with low sequence homology to Cry toxins [77]. Cyt toxins affect mainly mosquitoes that are vectors of human diseases; *Anopheles spp* (malaria), *Aedes spp* (dengue, zika, and chikungunya), and *Culex spp* (Nile fever and Rift Valley fever) [78]. Bt subsp. israelensis (Bti) is the most commonly used worldwide to control these vectors [3,78], because it produces Cry4Aa, Cry4Ba, Cry10Aa, Cry11Aa, Cyt1Aa, Cyt2Ba, and Cyt1Ca toxins that together act synergistically to kill mosquito larvae of Culex, Aedes, and Anopheles [79,80] (Table 1).

Furthermore, Cyt proteins have other important biologic targets, among others, mammalian and erythrocyte cells [81]; and the pea aphid (*Acyrthosiphon pisum*) and certain weevils (*Diaprepes abbreviates*) [82], both pest insects where Cyt toxins could be used as biocontrol.

Three subfamilies of Cyt toxins have been described so far, all of them sharing a high level of sequence identity: Cyt1 (1Aa, 1Ab, 1Ab, 1Ac, and 1Ad), Cyt2 (2Aa, 2Ba, 2Bb, 2Bc, and 2Ca), and Cyt3Aa1 [10]. Cyt1Aa shows significant similarity with volvatoxin 2 (VVA2), a cardiotoxin isolated from the mushroom *V. volvacea* [83]. VVA1 and VVA2 form the VVA toxin family, a PFT family that has hemolytic and cytotoxic activity in human red blood cells and tumor cells, respectively [84]. Cyt1Ca is different from other members of the Cyt family, since they have an extra domain in the C-terminal end with homology to a carbohydrate-binding domain of ricin. However, there are no reports of larvicidal or hemolytic activity for Cyt1Ca [11,85].

The overall structure of Cyt toxins is formed by a β sheet consisting of six antiparallel strands flanked by a α helix layer and an extra strand β0 at the N-terminal end, which could be involved in dimerization and proteolytic activation [85]. Notoriously, an extra strand at the N-terminal end has not been reported in Cyt2 toxins.

#### Cyt Proteins Mechanism of Action

Cyt proteins are synthesized as short protoxins [3,86]. The proteolytic cleavage sites in Cyt toxins are found at N-terminal and C-terminal ends [77]. It is widely recognized that loops of the helices are involved in membrane–cell interaction and intermolecular assembly [80]. However, the action mechanism followed by Cyt toxins is still not clear; moreover, it is unknown whether there are specific receptors through which Cyt toxins recognize their target cells. Nevertheless, there are currently two main models of the cytotoxicity mechanism carried out by Cyt toxins.

##### (a) The Pore-Formation Model

**Figure 3:**
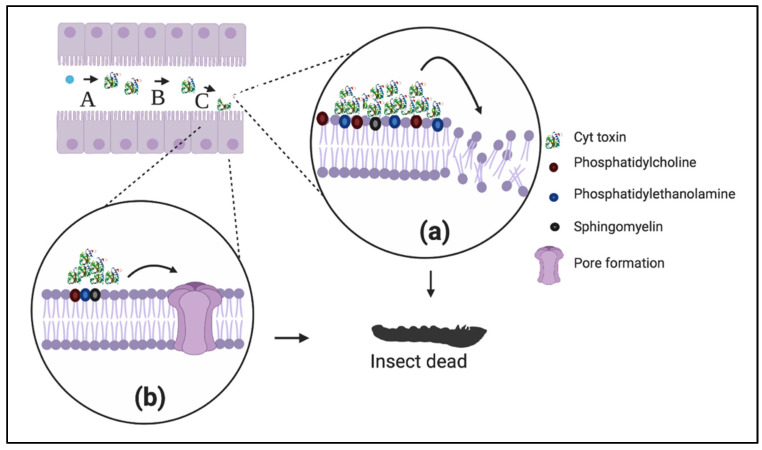
Mechanism of action of Cyt proteins. (**a**) Pore-forming model; once Cyt toxins interact with phosphatidylcholine, phosphatidylethanolamine, and sphingomyelin, they undergo a conformational change that helps recruit six Cyt monomers and assemble them into an open-umbrella structure, which results in a pore-formation and subsequent membrane permeabilization and larva death. (**b**) Detergent effect model; high Cyt toxin concentration binds to the lipid bilayer on cell membrane surface and destroys it through a detergent-effect. Both, the pore-formation model and detergent model are not mutually exclusive, because, the detergent effect acts at high Cyt toxins concentrations, while the pore-forming model acts at low Cyt toxins concentrations. A, Solubilization. B, Activation by proteolysis. C, Recognition of membrane receptor. Created with Biorender.com.

The pore-formation model describes binding of Cyt toxins in their monomer form to specific receptors in the membrane surface, similar to the Cry toxin mechanism. In this case, Cyt toxins interact directly with saturated membrane lipids such as phosphatidylcholine, phosphatidylethanolamine, and sphingomyelin [80,81]. Cyt toxins undergo a conformational change that helps to recruit six monomers of Cyt and assemble them into an open-umbrella structure in which the strands span the lipid bilayer transversely, while alpha helices rest on the membrane surface. The result is a pore-forming model with membrane permeabilization (Figure 3a) [80].

##### (b) The Detergent-Effect Model

The detergent-effect model suggest that Cyt toxins kill target cells through a solubilization effect on their membrane. In this model, Cyt toxins concentrate on the cell membrane surface and destroy the lipid bilayer in a detergent-like manner (Figure 3b) [80].

Both the pore-formation model and detergent model are not mutually exclusive, as it is thought that, depending on toxin concentration, one or both may act on susceptible cells [80]. Cyt oligomerization and pore formation could be carried out at low Cyt concentration [87], while the detergent effect could be induced only at high toxin concentration [87]. Therefore, the cell membrane from target cells is unable to assemble oligomers at high toxin concentration; instead, it forms a toxin–lipid complex in which the integrity of the membrane is completely lost [80].

Another mechanism of action of Cyt toxins involves a synergistic activity between different members of the family. Thus, when Cyt1Ab and Cyt2Ba act together, they enhance the insecticidal activity against *Aedes aegypti* larvae and resistant *Culex quinquefasciatus* larvae [88]. It has also been found that two known epitopes of Cyt1Aa (196EIKVSAVKE204 and 220NIQSLKFAQ228) binds to Cry4B and Cry11Aa toxins to enhance their toxic effect against the mosquito *Anopheles albimanus* and *Culex quinquefasciatus* [15,84,89]. Epitopes of Cyt1Aa play a receptors role to Cry4B and Cry11Aa, similar to the cadherin receptor of *Manduca sexta*. When Cyt1Aa binds to membrane cell receptors, Cry11Aa or Cry4B binds to this toxin, increasing oligomerization and pore formation effects [3,89,90]. Another synergistic effect occurs when Cyt toxins act together with Mtx1 (another toxin produced by Bt) against *Culex quinquefasciatus* larvae [91].

In recent years, several studies have been published related to Cyt proteins modification throughout genetic engineering to produce chimeric toxins [82,92]. These modifications take advantage of small Cyt toxin sizes and low degree complexity of their quaternary structure, as well as their high toxic capacity. The advantages of creating chimeras is to diversify their targets and to increase Cyt toxin potency.

The first successful report of a Cyt chimeric creation describes the insertion of a pea aphid gut-binding peptide GBP3.1, into the amino acid sequence of Cyt2Aa toxin. This peptide prevents the uptake of a plant virus by its vector, the pea aphid. It comprises 12 amino acids (TCSKKYPRSPCM), which bind to alanyl aminopeptidase-N on membrane surface of the aphid gut epithelium. Naturally, Cyt2Aa has low toxicity against the pea aphid, however, Chogule and coworkers succeeded in enhancing the binding of Cyt toxin and increasing its toxicity against aphids, by turning it into a chimeric protein [82].

In another study, Torres-Quintero et al. [92] modified Cyt1Aa by inserting the amino acid sequence of loop3 from Domain II of Cry1Ab (FRSGFSNSSVSI), which induces binding affinity of Cyt1Aa toxin to APN and CAD receptors of *Manduca sexta* [91]. Naturally, Cyt1Aa is not toxic to *M. sexta*, however, chimeric toxin had more significant toxicity to *M. sexta* and *Plutella xylostella* [92]. These results open new possibilities to the application of delta-endotoxins from *Bacillus thuringiensis*, to a new target pest.

### 2.3. Parasporins

Extensive screening analysis to find new possible targets to Bt strains has shown that non-insecticidal Cry proteins are more widely distributed in nature than insecticidal Cry proteins [16,93,94]. This fact has led researchers to inquire about the possible biological activities or targets of non-insecticidal and non-hemolytic Cry proteins. In this sense, Mizuki et al., in 1999, was the first group to report delta-endotoxins from Bt with selective cytotoxicity against leukemia cells, after a large-scale screening analysis involving protease-digested parasporal proteins from 1744 Bt strains. 1700 strains were isolated in Japan, while 44 were obtained from the Pasteur Institute in Paris [95]. From the isolated strains, 60 presented hemolysis activity and were eliminated by containing *cyt* genes, the rest of strains were tested in vitro for cytocidal activity against MOLT-4 cells and insecticidal activity [95]. At the end of the screening, authors selected only two strains (A1190 and A1462), because they produced toxins that selectively killed leukemic cells instead of normal T-cells [95]. These results inspired an intense and extensive screening of non-insecticidal and non-hemolytic toxins with cytotoxicity against cancer cells throughout the world, which led to the classification of a new type of Cry proteins called parasporins (PS) [96]. At first, this new classification included all non-insecticidal and non-hemolytic Cry toxins with selective cytotoxic activity against cancer cells [7,96]. Time later, it was accepted that non-hemolytic but insecticidal activity could also be part of this classification [96]. Bt strains that produce PS have been isolated from soils of various ecosystems in several countries such as Japan [95,97,98,99,100,101,102,103,104,105,106,107,108,109,110,111,112,113,114,115,116,117,118,119,120], Vietnam [121,122], India [94,123], Malaysia [124], China [125], Iran [126], and Saudi Arabia [127]. Canada [128] and Caribbean [129] have contributed with new parasporins.

In nature, there are several toxins produced by bacteria capable of killing mammalian cells through pores formation in cell membranes and/or by apoptosis activation [51]. Such is the case of aerolysin from *Aeromonas hydrophila* and alpha-toxin of *Clostridium perfringens*, which are both PFTs that recognize GPI-anchored proteins in the membrane of susceptible cells [51]. Some parasporins share structural homology with both toxins, therefore, it is assumed that PS proteins contain an action mechanism similar to aerolysin and alpha-toxin [130]. So far, 19 parasporins have been identified and organized in six families (Table 2) [7,96].

#### 2.3.1. Parasporin Classification

##### PS1 Family

PS1 family has cytotoxic effects against certain cancer cell lines such as HeLa (cervix cancer) [99], HL60 (leukemia) [109], Jurkat (leukemia), and HepG2 (liver cancer) [128]. Findings suggest PS1 toxins recognize a common receptor contained between these cell lines, identified as beclin-1 [131]. In healthy cells, beclin-1 exists intracellularly and is involved in autophagia and apoptosis processes, however, in susceptible cell, beclin-1 exists extracellularly and acts as a PS1 receptor [132].

The PS1 family includes, PS1Aa1 (Cry31Aa1), PS1Aa2 (Cry31Aa2), PS1Aa3 (Cry31Aa3), PS1Aa4 (Cry31Aa4), PS1Aa5 (Cry31Aa5), PS1Aa6 (Cry31Aa6), PS1Ab1 (Cry31Ab1), PS1Ab2 (Cry1Ab2), PS1Ac1 (Cry31Ac1), PS1Ac2 (Cry31Ac2) [96].

##### PS2 Family

PS2Aa1 (Cry46Aa1), PS2Aa2 (Cry46Aa2), and PS2Ab2 (Cry46Ab1) proteins constitute the PS2 family [96]. It is reported that the Bt A1547 strain produce PS2Aa1 and PS2Aa2 [95]. Parasporins from this family are produced as small toxins of around 30 kDa that are cytotoxic to cancer cell lines like HepG2 (liver cancer), Sawano (endometrial cancer), HL60, CaCo-2 (colon cancer), Jurkat (leukemia), and MOLT-4 [133,134].

A peculiarity of PS2 family toxins is that they do not have a typical 3d structure; instead, they are very similar to PFT-aerolysin which is formed mainly by elongated β sheets [133,134]. In 2009, Akiba et al. [134] proposed that PS2 toxins were able to produce oligomers that induce membrane pores and cell death by bind to lipid rafts. In 2017, Abe et al. reported that GPI was an essential co-receptor to PS2 parasporins toxic activity [135]. The action mechanism of PS2 family members is apparent by activating apoptosis, which is associated to an increase in the tumor suppressor gene PAR-4 expression and through inhibition of the PI3K/AKT pathway [136].

##### PS3 Family

PS3Aa1 (Cry41Aa1) is the single member of this family [96]. This parasporin is the only one with a ricin domain that plays a role in stabilization of the interaction between toxins and carbohydrate residues of the membrane [137]. In 2018, a research group led by Crickmore studied the PS3 protein and observed that it is structurally related to insecticidal toxins, except for the ricin domain. Using site-directed mutagenesis, they concluded that ricin domain is not associated with PS3 selective cytotoxic activity against the HepG2 cancer cell line [138].

In contrast to the mechanism of action used by PS2, it was proposed that PS3 induced cell death by necrosis throughout a pore formation in cancer cell membranes, as was evidenced by an increase in lactate dehydrogenase (LDH) release; mainly in HL60 and HepG2 cancer cell lines [138].

##### PS4 Family

PS4 is similar in size and structure to PS2 family members, its active form is around 27 kDa and presents a selective cytotoxicity against MOLT-4, CaCo-2, HL60, U937, HepG2, Sawano, DE-4 (leukemia), TS (uterine cancer), and TCS (cervical cancer) cancer cell lines [114,115]. A peculiarity of PS4 toxin is that it can be activated in both alkaline and acid pH [129], while most of parasporins are solubilized in alkaline pH. Actually, acid pH increases their cytotoxic effect against several cancer cell lines [115].

Regarding the mechanism of action of PS4, the evidence found (non-specific binding to the membrane, release of LDH and entrance into the cell of dextrans with different molecular weights) suggests that cell death occurs by necrosis [114,115,130]. The PS4 family includes PS4Aa1 (Cry45Aa1) as the only member [96].

##### PS5 Family

PS5Aa1 (Cry64Aa1) is the only member of the PS5 family; this protein has been isolated from the *Bt tohokuensis* A1100 strain [96]. The active form of PS5Aa1 has an approximate size of 30 kDa and is selectively toxic to MOLT-4 [120]. Concerning sequence similarity, PS5 shows higher similarity to PFT aerolysin than the other parasporins [120,139]. It has been reported that PS5 is toxic to MOLT-4, Jurkat, HL-60, HepG2, HeLa, Sawano, TCS, CaCo-2, and K562 cancer cell lines, but it also shows potent activity against healthy tissue cells such as UtSMC (normal uterus) and MRC-5 (normal lung) [120]. However, there is still no evidence about its mechanism of action.

##### PS6 Family

PS6 is closely related to PS1, sharing a conserved sequence of fifty amino acids. PS6 is selectively toxic to HepG2 and HeLa [117], but its mechanism of action is still unknown. The PS6 family includes only PS6Aa1 (Cry63Aa1), which has been isolated from Bt M019 [96,117] and 64-1-94 [129].

#### 2.3.2. Mechanism of Action of Parasporins 

The cytocidal activity of PS against cancer cell lines ranges from EC_50_ 0.0017 µg/mL of PS2 against Sawano to 3.0 µg/mL of PS1 against HepG2. Table 2 shows the reported EC_50_ to PS in cancer cell lines.

In management of parasporins, different methods of crystals solubilization and activation should be tried, since effectiveness of their cytocidal activity against cancer cells depends on this. Similar to what happens on insects, a correct activation of protoxins is essential for cell membrane receptors recognition and subsequent triggering of cancer cell death.

As an example, there is a particular case of PS2Aa1 not showing toxic activity when activated using trypsin, but, when activated with proteinase K, it shows activity against human cancer cells [137]. In this sense, it is crucial to know that sites of cleavage to trypsin and proteinase K are different.

The mechanism of action of parasporins against target cancer cells is poorly understood, however, available information has shown that parasporins exhibit several mechanisms of action to kill cancer cells. These proteins act in a similar way to Cry toxins because they are highly specific to a cell type, nevertheless, it is well known that Cry toxins specificity depends on cell membrane receptors (cadherin, aminopeptidase-N, and alkaline phosphatase) recognition. On the other hand, PS interaction with cell membrane receptors is still being investigated, several molecules that act as parasporin receptors have been reported and patented. In this sense, Beclin-1 acts as PSAa1 receptor [131]. Glycosylphosphatidylinositol (GPI)-anchored protein is involved in efficient cytocidal action of PS2Aa1 [140]. GADPH from CEM-SS leukemic cell line acts as receptor from a PS found in Malaysia [124]. One of the most important characteristics of parasporins is their ability to discriminate cancer cells from non-cancer cells, which is directly related to cell membrane receptors recognition.

### 2.4. S-Layer Proteins

Surface layer proteins (SLP) are widely represented, both in Gram-negative and Gram-positive bacteria, including *Bacillus* [17]. Similar to delta-endotoxins, SLP are assembled into parasporal positions with several shapes (oblique, square, or hexagonal) [8]. They have a molecular mass between 40 and 170 kDa [141,142], and are involved mainly in growth, survival, and maintenance of cell integrity [9]. There are also reports of their antiviral and antibacterial activity, as well as of their anti-inflammatory effects [141,142].

SLP toxins activity is still unclear; it has been suggested that SLP have a similar insecticidal activity to Cry proteins but with a different mechanism [141,142]. The SLP obtained from GP1 Bt strain is the only one that has been reported to have pesticidal activity against *Epilachna varivestis* [18].

A recent study reported an SLP from Bt with high selective cytotoxic activity in vitro against the MDA-MB-231 breast cancer cell line. Authors suggest that cadherin-11 receptor present in cancer cells seems to be involved in SLP recognition; however, the mechanism of action is still under study [8].

### 2.5. Toxins Secreted by Bt

In addition to Cry, Cyt, PS, and SLP toxins produced in parasporal bodies during sporulation, Bt secretes during vegetative growth phase other toxins with insecticidal activity [11]. There are two main families of secreted insecticidal proteins, one is known as vegetative insecticidal proteins (Vip) [20,21] and the other as secreted insecticidal proteins (Sip) [19]. These proteins contain a signal peptide sequence in the N-terminal end that is cleaved after the secretion process is completed [143,144].

#### 2.5.1. Vip Family

Vip toxins are not shaped as parasporal inclusion bodies, instead they are produced and secreted during the vegetative growth phase and their expression ends before the sporulation stage begins [11]. These insecticidal toxins have been characterized as; Vip1, Vip2, Vip3, and Vip4; however, their mechanism of action against insects is not entirely understood yet [20,28].

Vip1 is synthesized as a protoxin of 100 kDa and after secretion; a mature toxin of 80 kDa is produced, additionally, Vip2 releases a trypsin-resistant fragment of 50 kDa [144]. Together, Vip1 and Vip2 produce a Vip binary toxin [145], with synergistic insecticidal activity against some coleopteran pests and the sap-sucking insect pest *Aphis gossypii (Hemiptera)* [146].

Vip2 is similar in structure and behavior to the CdtA toxin from *Clostridium difficile*; this toxin presents an ADP-ribosyltransferase activity and its principal target is the actin protein, therefore, could induce cytoskeletal disruption and cell death when it is activated [147].

In monomer form, Vip1 binds to its receptors and a conformational change is produced because of this interaction; thus, more Vip1 toxins are attracted to form a heptamer that translocates Vip2 into the cytoplasm through acid endosomes [148]. Once inside the cell, Vip2 destroys actin filaments that disrupt the cytoskeleton and eventually induce cell death [148].

Due to its similarity with other A+B binary toxins, it has been concluded that Vip2 is responsible for most of the cytotoxic activity, while Vip1 is responsible for binding to membrane receptors in susceptible insects [149].

Vip3 is a single-chain protein that is toxic to a wide variety of lepidopterans and other insects, such as *Agrotis ipsilon*, *Spodoptera exigua*, and *S. frugiperda*, which are less susceptible to Cry1A toxins [21]. Vip3 are proteins of 88 kDa approximately without homology to any other known insecticidal protein [86,150]. In contrast to Vip1 and Vip2, signal peptide sequences in Vip3 are not processed during secretion and are present in the mature secreted peptide, suggesting they play an important role in protein structure and insecticidal activity. However, cleavage of the N-terminal end activates the protoxin; the 66 kDa active toxin is fragmented from the 22-kDa N-terminal portion [21]. The mechanism of action is still unclear and it has been suggested that Vip3 proteins act in a similar way to PFT, but their membrane receptors are still unknown. In vitro experiments have shown that Vip3 does not compete for binding sites of Cry1A in *Manduca sexta* nor *S. frugiperda* [151].

Vip 4 is the most recently discovered member of the Vip family. It has a molecular mass of ~108 kDa and a 34% identity to Vip1Aa1 protein, specifically to the B component of the binary toxin. For this reason, it has been suggested that Vip4 might interact with unknown A component to produce toxicity; therefore, such information is needed to understand its action mechanism [20].

The Vip proteins that have been reported so far are 15 Vip1 proteins, 20 Vip2 proteins, 101 Vip3 proteins, and 1 Vip4 [13].

#### 2.5.2. Sip Toxins

Secreted insecticidal proteins (Sip) are toxins produced by Bt with an approximate size of 41 kDa. Similarly to Vip proteins, Sip toxins are synthesized containing a signal peptide sequence of 30 amino acids, which is processed by proteases [19] and an active protein is released [11]. It is known that Sip proteins have insecticidal activity against Coleopterans such as *Leptinotarsa decemlineata*, *Diabrotica undecimpunctata howardi*, and *Diabrotica virgifera virgifera* [19]. However, their mechanism of action is still unknown.

## 3. Conclusions

The horizontal transfer of genetic information through the conjugation of plasmids in *Bacillus thuringiensis* opens up a world of possibilities for the discovery of new toxins, new structures, new targets, and even new classification.

Understanding the structural characteristics of Bt toxins and their mechanism of action will allow us to develop new products for improving pest management and human health. In this sense, new combinations of insecticidal Cry proteins have been recently found, opening new possibilities to pest control without genetic and neither molecular manipulation [152]. Additionally, in 2019, Mendoza and coworkers for first time reported that Cry1A toxins from Bt presented a highly specific anticancer activity in HeLa cells and also against insects. Authors suggested that in both cases, a specific interaction between Cry toxins and cell membrane receptors could be initiating toxicity on insects and in human cancer cells [153].

SLP proteins are other Bt toxins with both pesticide and anticancer activity. Recently studies have shown that these proteins also could be recognizing specific cell membrane receptors in cancer cells line [8], as Cry toxins do [153]. In addition to cytotoxic activity on insects and human cancer cell lines, SLP carry out structural and protection activities in several microorganisms. Furthermore, these proteins could be found in Gram-positive, Gram-negative, and archaebacteria, not only in Bt.

Parasporins also present specific anticancer activity in vitro; these proteins are found in non-insecticidal and non-hemolytic strains, opening the possibility to develop anticancer agents with therapeutic potential and without secondary effects in patients. However, very little is known about their mechanism of action and the receptors recognized to carry out their cytotoxicity. PS1 and PS2 are the most extensively studied parasporin families, therefore, have been used as a model to answers several questions regarding preferential activity of these toxins against cancer cells.

Finally, it is important to mention that more research is needed to understand the mechanisms of action used by Bt toxins. According to reported studies, it seems that most of them recognize specific cell membrane receptors in susceptible cells; however, what is happening inside cells once the interaction has begun still is a mystery. Thus, it is essential to know and understand the signaling pathways involved in toxicity to be capable of developing new anticancer compounds and to improve pest control including resistance developed to Bt toxins.

## Figures and Tables

**Table 1:** Cyt toxins could synergize with several Cry toxins to act against mosquitoes.

*Aedes spp.*	Culex spp.	Anopheles spp.
Cyt1Aa, Cyt2Ba	Cyt1Aa, Cyt2Ba	Cyt1Aa, Cyt2Aa
Cry4Aa	Cry4Aa	Cry4Aa
Cry4Ba	Cry4Ba	Cry10Aa
Cry10Aa	Cry10Aa	Cry11Aa
Cry11Aa	Cry11Aa	

**Table 2:** Parasporin characteristics.

PS	Bt Strain	Cry Gene	Protoxin (kDa)	Active Toxin (kDa)	Protease Activation	Main Cellular Target	EC50 [µg/mL]	Mechanism Action	Country	Reference
PS1Aa1	A1190	Cry31Aa1	81	15, 56	Trypsin	HeLa	0.12	Apoptosis	Japan	[99]
PS1Aa2	M15	Cry31Aa2	83	55, 70	Trypsin	Jurkat HepG2	0.02 0.02	ND	Canada	[128]
PS1Aa3	B195	Cry31Aa3	81	56	Trypsin	HeLa	14.7	ND	Japan	[116]
PS1Aa4	Bt79-25	Cry31Aa4	81	NP	Proteinase K	ND	ND	ND	Vietnam	[122]
PS1Aa5	Bt92-10	Cry31Aa5	81	NP	Proteinase K	ND	ND	ND	Vietnam	[122]
PS1Aa6	M019, 64-1-94	Cry31Aa6	70	15, 55	Trypsin	HepG2	0.52	ND	Japan, Caribbean	[117,129]
PS1Ab1	B195	Cry31Ab1	82	56	Trypsin	HeLa	14.7	ND	Japan	[116]
PS1Ab2	Bt31-5	Cry31Ab2	82	NP	Proteinase K	ND	ND	ND	Vietnam	[122]
PS1Ac1	Bt87-29	Cry31Ac1	87	NP	Proteinase K	ND	ND	ND	Vietnam	[122]
PS1Ac2	B0462	Cry31Ac2	81	15, 60	Proteinase K	HeLa	2	Apoptosis	Japan	[118]
PS1Ad1	64-1-94, M15, M019	Cry31Ad1	73	14, 59	Trypsin	HepG2	0.52	ND	Caribbean, Canada, Japan	[117,128,129]
PS2Aa1	A1547	Cry46Aa1	37	30	Proteinase K	HepG2	0.023	Pore-forming	Japan, USA	[105]
PS2Aa2	A1470	Cry31Aa2	30	28	Proteinase K	MOLT-4	0.041	ND	Japan	[119]
PS2Ab1	TK-E6	Cry31Ab1	33	29	Proteinase K	Jurkat	0.545ng/ml	ND	Japan	[113]
PS3Aa1	A1462	Cry41Aa1	88	64	Proteinase K	HL60	1.32	ND	Japan	[100]
PS3Ab1	A1462	Cry41Ab1	88	64	Proteinase K	HL60	1.25	ND	Japan	[100]
PS4Aa1	A1470	Cry45Aa1	31	28	Proteinase K	CaCo2	0.124	Pore-forming	Japan	[111]
PS5Aa1	A1100	Cry64Aa1	33	30	Proteinase K	TCS	0.046	ND	Japan	[120]
PS6Aa1	M019, 64-1-94	Cry63Aa1	85	14, 59	Trypsin	HepG2	2.3	ND	Japan, Caribbean	[117,129]

NP- not published. ND- not determined.
